# Volatile Organic Compounds Emitted by *Aspergillus flavus* Strains Producing or Not Aflatoxin B1

**DOI:** 10.3390/toxins13100705

**Published:** 2021-10-06

**Authors:** Laurie Josselin, Caroline De Clerck, Marthe De Boevre, Antonio Moretti, M. Haïssam Jijakli, Hélène Soyeurt, Marie-Laure Fauconnier

**Affiliations:** 1 Laboratory of Chemistry of Natural Molecules, Gembloux Agro-Bio Tech, Liege University, Passage des déportés 2, 5030 Gembloux, Belgium; marie-laure.fauconnier@uliege.be; 2 AgricultureIsLife, Gembloux Agro-Bio Tech, Liege University, Passage des déportés 2, 5030 Gembloux, Belgium; Caroline.declerck@uliege.be; 3 Centre of Excellence in Mycotoxicology and Public Health, Department of Bioanalysis, Faculty of Pharmaceutical Sciences, Ghent University, Ottergemsesteenweg 460, 9000 Gent, Belgium; Marthe.DeBoevre@UGent.be; 4 Institute of Sciences of Food Production, National Research Council, Via Amendola 122/o, 70126 Bari, Italy; antonio.moretti@ispa.cnr.it; 5 Integrated and Urban Plant Pathology Laboratory, Gembloux Agro-Bio Tech, Liege University, Passage des déportés 2, 5030 Gembloux, Belgium; mh.jijakli@uliege.be; 6 Statistic, Informatic and Applied Modelling, Gembloux Agro-Bio Tech, Liege University, Passage des déportés 2, 5030 Gembloux, Belgium; hsoyeurt@uliege.be

**Keywords:** aflatoxin B1 (AFB1), *Aspergillus flavus*, microbial volatile organic compounds (mVOCs), solid phase microextraction (SPME), toxigenic, terpenes, mycotoxins, semi-quantification

## Abstract

*Aspergillus flavus* is a phytopathogenic fungus able to produce aflatoxin B1 (AFB1), a carcinogenic mycotoxin that can contaminate several crops and food commodities. In *A. flavus*, two different kinds of strains can co-exist: toxigenic and non-toxigenic strains. Microbial-derived volatile organic compounds (mVOCs) emitted by toxigenic and non-toxigenic strains of *A. flavus* were analyzed by solid phase microextraction (SPME) coupled with gas chromatography–mass spectrometry (GC-MS) in a time-lapse experiment after inoculation. Among the 84 mVOCs emitted, 44 were previously listed in the scientific literature as specific to *A. flavus*, namely alcohols (2-methylbutan-1-ol, 3-methylbutan-1-ol, 2-methylpropan-1-ol), aldehydes (2-methylbutanal, 3-methylbutanal), hydrocarbons (toluene, styrene), furans (2,5-dimethylfuran), esters (ethyl 2-methylpropanoate, ethyl 2-methylbutyrate), and terpenes (epizonaren, trans-caryophyllene, valencene, α-copaene, β-himachalene, γ-cadinene, γ-muurolene, δ-cadinene). For the first time, other identified volatile compounds such as α-cadinol, cis-muurola-3,5-diene, α-isocomene, and β-selinene were identified as new mVOCs specific to the toxigenic *A. flavus* strain. Partial Least Square Analysis (PLSDA) showed a distinct pattern between mVOCs emitted by toxigenic and non-toxigenic *A. flavus* strains, mostly linked to the diversity of terpenes emitted by the toxigenic strains. In addition, the comparison between mVOCs of the toxigenic strain and its non-AFB1-producing mutant, coupled with a semi-quantification of the mVOCs, revealed a relationship between emitted terpenes (β-chamigrene, α-corocalene) and AFB1 production. This study provides evidence for the first time of mVOCs being linked to the toxigenic character of *A. flavus* strains, as well as terpenes being able to be correlated to the production of AFB1 due to the study of the mutant. This study could lead to the development of new techniques for the early detection and identification of toxigenic fungi.

## 1. Introduction

Contamination by filamentous fungal species such as *Aspergillus, Fusarium,* and *Penicillium* in different types of agricultural commodities such as grains is a common phenomenon [1,2]. Many species of these genera have the ability to produce small, non-volatile, secondary metabolites, namely mycotoxins, which are (possibly) harmful for humans and more generally to all vertebrates, even at low concentrations. These fungi have the ability to produce mycotoxins during pre- or post-harvest conditions, and could possibly exert adverse health effects upon dietary consumption by both animal and humans. Besides the latter, the damage of fungi on the agricultural crop leads to residual crop and subsequent trade loss for the agricultural entrepreneurs [3,4,5]. In particular, many species of *Aspergillus* can colonize cereals [6], including *Aspergillus flavus,* which occurs frequently on maize at different stages of both pre-harvest and post-harvest conditions [7,8]. The *A. flavus* species includes toxigenic strains producing mycotoxins and non-toxigenic strains not producing mycotoxins. Their difference is linked to the presence of a gene in the toxigenic strains that gives them the ability to produce aflatoxins [9,10]. The main mycotoxin produced by *A. flavus* is aflatoxin B1 (AFB1) [11]. Studies have shown that human chronic exposure to AFB1 may lead to hepatocellular cancer, and that a single acute exposure could result in the death of the consumer [12,13].

The determination of the aflatoxins content in cereals is commonly performed using liquid chromatography coupled to mass spectrometry (LC-MS/MS), however, under certain conditions, such as in the field, rapid immuno-chromatographic competitive assay strips are used to enable a fast decisive result [14,15]. When applying quantitative LC-MS/MS procedures, an extensive sample clean-up is required, and the analysis itself is expensive, complex, and requires trained staff. These tests are therefore not suitable to analyze large numbers of samples in, for example, a remote setting where fast results are required [16,17,18,19]. 

Several scientific reports have already shown that there is a correlation between the occurrence of certain volatile organic compounds (VOCs) and the presence of fungi in foodstuffs [20,21], as well as in indoor buildings [22,23]. These VOCs have been referred to as microbial VOCs (mVOCs) [24]. Even if mycotoxins are not volatile contaminants, we hypothesize that their production is possibly linked to the emission of mVOCs, produced through common parts of biosynthetic pathways linked to the mycotoxin production [10]. Citron et al. highlighted the correlation between the secondary metabolism of *Actinomycetes* that are rich in terpenoids and their genome [25]. Keller et al. studied the synthesis of molecules from the secondary metabolism of fungi and the production of toxins [26].

In *A. flavus*, not all strains have the same toxigenic potential, it is common to isolate and identify strains that produce AFB1 and strains that do not. These latter strains are called non-toxigenic strains and, since they lack different genes of the AFB1 biosynthetic gene cluster, are used as biological control agents on several crops to reduce the incidence of AFB1-producing strains [27].

The objective of this work is to determine if specific mVOCs are emitted when mycotoxins are produced in the setting of *A. flavus* strains, with the final aim of developing rapid online detection systems. These specific mVOCs could allow a faster and indirect detection of AFB1 produced by *A. flavus*. In this study, we have analyzed the mVOCs emitted by non-toxigenic and toxigenic strains of *A. flavus*. In addition, we have compared the emission of a toxigenic strain producing AFB1 (ITEM 8111) with its non-aflatoxigenic mutant (ITEM 8111*).

## 2. Results

**Figure 1:**
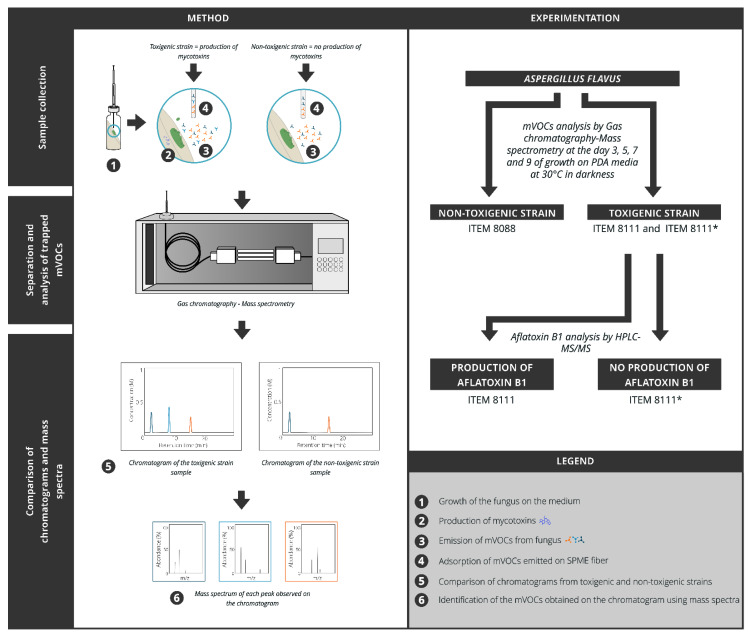
General outline of the experiment and the methods used.

The results are derived from the detection of mVOCs emitted at different days of fungal growth of the three strains studied, as well as their AFB1 concentration. The method and experimentation are summarized in Figure 1.

### 2.1. Aflatoxin Concentrations

AFB1 concentrations in the toxigenic AFB1-producing strain (ITEM 8111) were: 70.30 µg·kg^−1^ on day 3; 82.20 µg·kg^−1^ on day 5; 2321.60 µg·kg^−1^ on day 7; and 149.20 µg·kg^−1^ on day 9 after inoculation. AFB1 concentrations in the toxigenic non-aflatoxin producing strain (ITEM 8111*) were below the limit of quantification (<3 µg·kg^−1^).

As expected, no aflatoxins were detected in the non-toxigenic samples (ITEM 8088).

### 2.2. mVOCs Screening

The 84 compounds identified to be emitted by the three *A. flavus* strains (35 terpenes, 3 ketones, 2 furans, 4 alkenes, 9 alkanes, 4 aldehydes, 11 alcohols, 6 esters, 2 acids, 2 others, and 6 non-identified) are listed in Table 1.

**Figure 2:**
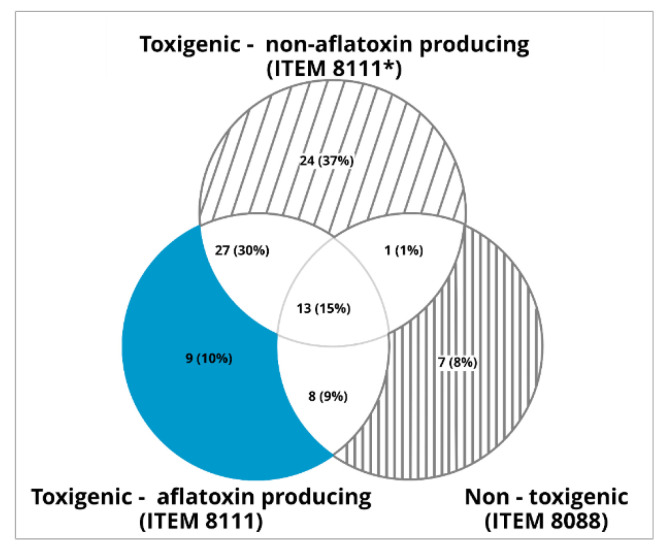
Number of compounds (and their percentage) emitted only by one of the three strains and compounds common to two or three strains.

We observed that the non-toxigenic strain emits a smaller number of mVOCs than the toxigenic strains. A total of 22 mVOCs common to toxigenic and non-toxigenic strains were released (Figure 2).

Ethanol, 2-methylpropan-1-ol, 2-methylbutan-1-ol, 3-methylbutan-1-ol, and propan-1-ol were predominant for each day studied with similar values for each strain. Some hydrocarbons (2,2,4,6,6-pentamethylheptane, hexane, heptane, styrene), aldehydes (2-methyl-2-butenal, 2-methylbutanal, 3-methylbutanal), and esters (ethyl isobutyrate, ethyl propanoate, ethyl acetate, ethyl 2-methylbutyrate) were also detected in common, as well as 2,5-dimethylfuran, 3-hydroxybutan-2-one, 2,4,5-trimethyl-1,3-dioxolane, trichloromethane, and a single terpene (β-himachalene) (Table 1).

Some compounds were specifically and punctually emitted by the non-toxigenic strain (ITEM 8088):

- On day 7: (E,Z)-1,2-diethylidenecyclopentane, NI 640, NI 729, NI 756;
- On day 9: propan-2-ol with a large relative area of 72.3%;
- Sno specific compound emission is recorded on day 3 and on day 5.

Interestingly, with the exception of β-himachalene emitted punctually on day 3, no terpene emission was detected during the 9 days of analysis for the non-toxigenic strain.

**Figure 3:**
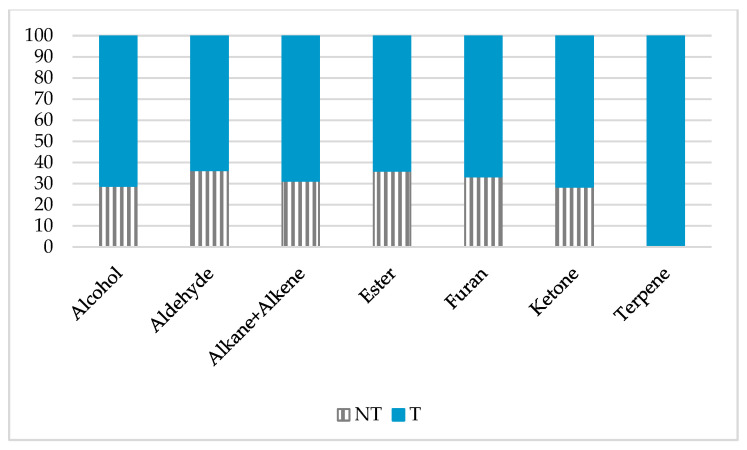
Distribution of the relative proportion of each family of molecule (NT: non-toxigenic, T: toxigenic). The percentage of non-toxigenic (NT) for the terpenes is less than 1%, so it is not visible on this graph.

The main difference that characterizes the toxigenicity of the strains is the abundance of terpenes emitted by the toxigenic strains (Figure 3).

In the case of the toxigenic strains (ITEM 8111 and ITEM 8111*), 60 different mVOCs have been identified, among which 27 as β-cadinene or viridiflorol were emitted in common (Figure 1).

For both strains, we observed a similar punctual emission on day 3 of decan-1-ol, 2-butyloctan-1-ol, aromadendrene, epi-cubeno-1-ol, palustrol, trans-caryophyllene, viridiflorol, α-calacorene, α-copaene, α-cubebene, and β-cadinene.

Unlike in the non-toxigenic strain, a constant emission of epizonaren and δ-cadinene was recorded.

Like in ITEM 8088, styrene was detected on day 3, but unlike in the non-toxigenic strain, where emissions were punctual, styrene emissions in the strain ITEM 8111 producing AFB1 persisted during the 9 day period considered. 

In the AFB1- producing strain (ITEM 8111), 2 -methylbutanal and 2-methylbut-2-enal were continuously emitted, while they were emitted only on day 3 by the non-AFB1-producing strain (ITEM 8111*). Cis-muurola-3,5-diene, germacrene-D, α-cadinene, α-gurjunene, α-isocomene, β-elemene, and γ-cadinene were emitted more or less regularly by the two toxigenic strains.

Interesting differences were spotted between the two toxigenic strains. Several molecules (heptadecane, γ-gurjunene, epi-bicyclosesquiphellandrene, and α-selinene) were punctually emitted (usually on day 3) for the AFB1-producing strain (ITEM 8111), while the emissions persist in time for the non-AFB1-producing strain (ITEM 8111*).

Butan-2,3-diol, nonyl-cyclopropane, 4,6-dimethyldodecane, octane, toluene, ethyl butyrate, NI 1323, and 2-methylfuran were only detected for the AFB1-producing strain (ITEM 8111). 

Hydrocarbons (methyl-cyclooctane, dodec-1-ene, bicyclo[2.2.0]hexa-2,5-diene), one ester (ethyl benzeneacetate), one ketone (butan-2-one), one alcohol (butan-1-ol), terpenes (4a,8-dimethyl-2-(prop-1-en-2-yl)-1,2,3,4,4a,5,6,7-octahydronaphthalene, (7a-Isopropenyl-4, 5-dimethyloctahydroinden-4-yl)methanol, di-epi-1,10-cubenol, valencene, α-corocalene, β-chamigrene, τ-muurolol), and other (including unidentified) compounds (thiochroman-4-one, NI 1271, NI 1501) were only detected for the non-AFB1-producing mutant of strain ITEM 8111.

**Figure 4:**
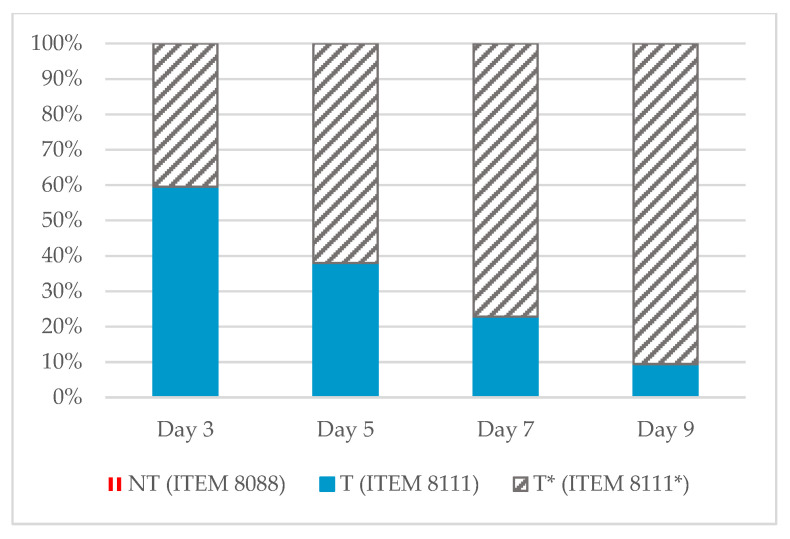
Terpenes distribution between the strains (NT: non-toxigenic strain, T: toxigenic aflatoxin B1 (AFB1)-producing strain, T*: toxigenic non-AFB1-producing strain) emitted of each time point (day 3, 5, 7, 9). The percentage of non-toxigenic (NT) is only present for day 3 and less than 1%, so it is not visible on this graph.

In comparison with the other strains, AFB1-producing strain (ITEM 8111) has the lowest terpene diversity. These terpenes emissions decreased over time until their total absence at day 9. In addition, the total number of terpenes emitted by the non-AFB1-producing mutant of strain ITEM 8111 was higher than for the AFB1-producing strain (ITEM 8111), on all days considered (Figure 4). 

**Figure 5:**
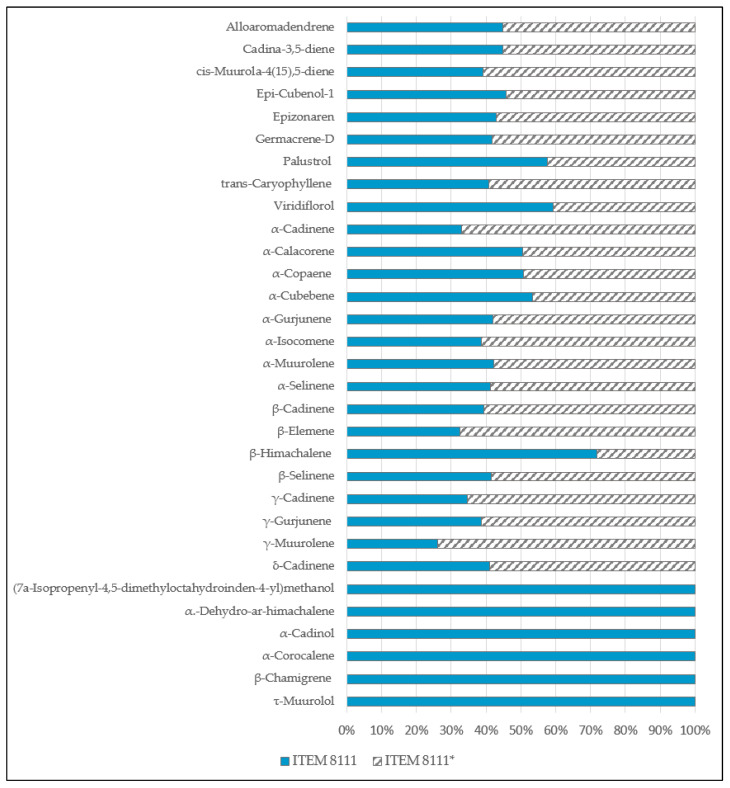
Proportion (%) of terpenes released during day 3 by the toxigenic AFB1-producing (ITEM 8111) and the toxigenic non-AFB1-producing (ITEM 8111*).

The majority of terpenes’ highest emissions were detected at the 3^rd^ day. Among the 32 terpenes emitted, 26 were in common and were emitted in similar proportions in both toxigenic strains. However, 6 compounds were specific to the non-AFB1-producing mutant of strain ITEM 8111 (Figure 5).

### 2.3. mVOCS Related to Toxigenic Characteristic

**Figure 6:**
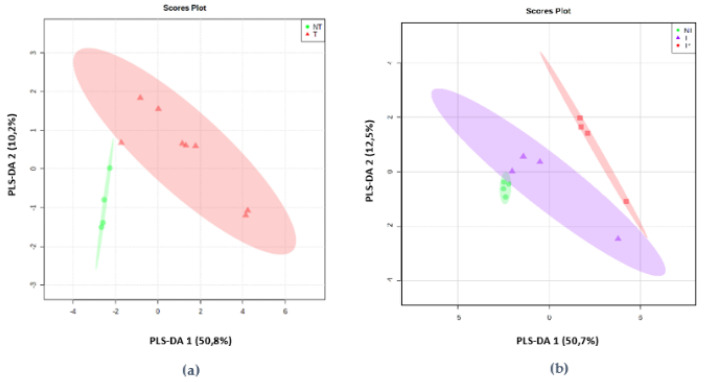
PLSDA (Partial Least Square Analysis) applied on the data (**a**) of the toxigenic (T-∆) and non-toxigenic (NT-○) strains, (**b**) of the AFB1-producing (T-∆), the non-AFB1-producing (T*-□), and non-toxigenic (NT-○).

Partial Least Square Analysis (PLSDA) shows the presence of a split according to the toxigenicity of the strain (Figure 6a). This division is mainly related to the terpenes emitted by the toxigenic strains. The indicator molecules that can be used for toxigenicity are epizonarene, δ-cadinene, germacrene-D, β-himachalene, γ-cadinene, β-selinene, γ-gurjunene, α-isocomene, and α -cadinene. Ethyl 2-methylbutyrate and heptane can be linked with the non-toxigenic strain.

Notable discrepancies were confirmed in the group of the toxigenic strain and its mutant (Figure 6b). Indeed, styrene, β-selinene, and γ-gurjunene emissions separated the AFB1-producing strain (ITEM 8111) and the non-AFB1-producing strain (ITEM 8111*). 

For the most interesting molecules, identified through Table 1 and PLSDA, concentrations were determined on day 3, in order to emphasize qualitative as well as quantitative differences (Table 2). 

## 3. Discussion

### 3.1. mVOCs

In our study, we have identified 57 compounds already known to be emitted by fungi (Table 1). In particular, we have identified 13 compounds known to be associated with the fungal presence (a in Table 1) and/or the genus *Aspergillus* (b in Table 1), and more precisely, 44 compounds known in the literature to be involved with the presence of *A. flavus* strains (c in Table 1) [10,23,28,29,30,31,32,33,34,35,36,37,38,39,40,41,42]. In addition, 20 compounds (not counting the 6 unidentified compounds) were identified for the first time to be emitted by *A. flavus.*

Among them: 2-butyloctan-1-ol, α-cadinene, α-calacorene, cis-muurola-3,5-diene, α-cubebene, α-selinene, β-cadinene, epi-cuben-1-ol, palustrol, viridiflorol, and α-isocomene, known to be emitted by fungi, were listed for the first time as specific volatiles of toxigenic strains of *A. flavus* (Table 1).

In addition, one new compound was systematically detected, in all strains and in significative proportions: 2-methylbut-2-enal, which was known to be emitted only from non-toxigenic strains, as per De Lucca et al. [42]. 

Like Sun et al. [31], we have observed that, unlike other chemical families, all strains emit the same alcohol proportions, whether toxigenic nor non-toxigenic.

The main difference between the toxigenic and the non-toxigenic strains was the presence/absence of terpenes (Figure 3). This correlation was already suggested in another study [32]. The terpenes identified are exclusively sesquiterpenes.

Terpenes are known to play several roles in nature. In fungi, they have been found to attract certain worms to defend them (trans-caryophyllene), to repel herbivores (trans-caryophyllene, α-muurolene, γ-muurolene) [35], and to be involved in inter- and intraspecific communication [43,44].

We observed that terpenes were only emitted in the case of the toxigenic strain and its mutant. Interestingly, these emissions tend to be continuous over time in the case of the non-AFB1-producing strain, while they are punctual (mainly on day 3) in the AFB1-producing strain. 

Several studies have already shown that the toxigenicity of *A. flavus* could be associated with punctual emissions of terpenes, like trans-caryophyllene, α-gurjunene, α-muurolene, and γ-muurolene (that we detected in our study on day 3) [23,30,31,32,33,34,35], and with constant emissions of epizonaren, γ-cadinene, and γ-gurjunene [29,31,32,36], which we detected during the 9 days of growth. These compounds were not listed in the literature as being emitted by a non-toxigenic *A. flavus* strain. This was not the case with δ-cadinene and valencene, which were detected in our study only in the toxigenic strain and its mutant, although they have been detected in the non-toxigenic strain in other studies [30,32,33].

Interestingly, we detected the presence of β-selinene and α-selinene, which are known to be precursors to the presence of mycotoxins [23]. As with other terpenes, these compounds are only emitted by toxigenic strains. However, we observed different patterns of emission between the toxigenic strain 8111 and its non-AFB1-producing mutant: punctual emission (at day 3) for the AFB1-producing strain and continuous emission during the 9 days for the mutant strain (ITEM 8111*). We also observed this emission profile for β-himachalene, γ-cadinene, germacrene-D, α-gurjunene, and epi-bicyclosesquiphellandrene, suggesting that these compounds could, in the same way, be involved in the toxin production.

Terpenes could also act as inhibitors of AFB1 synthesis, as was shown in Holmes et al. [45]. In our study, six terpenes are specifically emitted by the non-AFB1-producing mutant strain (ITEM 8111*) and could act as inhibitors. Among them, α.-dehydro-ar-himachalene, τ-muurolol, and α-cadinol, present in some essential oils, have shown antimicrobial and/or fungicidal activities [46]. However, whether such production of terpenes was the cause of the lack of AFB1 synthesis or was triggered by this loss of mycotoxin production needs to be better evaluated. 

In fungi, aflatoxins are supposed to be involved in defense against other external pathogens (bacteria, fungi, etc.) but also host-related defenses. In our non-AFB1-producing mutant strain (ITEM 8111*), the absence of AFB1 production could be compensated by an important and continuous emission of terpenes, playing similar roles. 

Other interesting compounds were detected. (E,Z)-1,2-diethylidenecyclopentane was only emitted by the non-toxigenic strain and is a known compound of *Laurus nobilis* essential oil, which has shown antifungal activities and caused inhibition of AFB1 in vitro [46]. The thiochroman-4-one emitted by the non-AFB1-producing strain was known to be an antifungal agent involved in population regulation [47]. 

Ethyl 2-methylbutyrate is the only volatile that can be related to the absence of AFB1 production for both non-toxigenic and toxigenic non-AFB1-producing strains. It has been identified as specific to the genus *Aspergillus* [46].

### 3.2. Potential mVOCs Markers

Several studies have already considered the use of mVOCs as potential biomarkers to detect the presence of fungi [48] and even mycotoxin contamination [49]. 

However, this kind of dispositive for the detection of *A. flavus* is not available yet, to the best of our knowledge. 

Our study provides, for the first time, a group of potential marker molecules that could be considered to determine the presence of *A. flavus* and its AFB1 production. 

Based on our results, some volatiles emitted in significant proportions, like 3-methylbutan-1-ol and 2-methylbutanal, could be used to detect the presence of a fungal contamination. Other volatiles like 2-methylbut-2-enal, ethyl isobutyrate, ethyl acetate, and δ-cadinene are specific to *A. flavus* and can be used to detect a specific contamination by this fungus.

More interestingly, some volatile compounds can be used to specifically detect the presence of *A. flavus* toxigenic strains. Among them, epizonaren is a good candidate, as it is emitted in significant proportion (5 ppb) continuously on every day of growth only by toxigenic strains. In other studies, this compound was already used as a fungal indicator [36] related to *A. flavus* [34] and has been detected for several *A. flavus* toxigenic strains [46].

Heptadecane, 2-methylfuran, and toluene were only detected for the toxigenic strain and could also be used as potential biomarkers. These compounds are already known as common fungal volatiles and used as indicators of fungal growth [30,39].

We did not show any mVOCs related to AFB1 production but rather to the absence of production in the non-AFB1-producing strain (ITEM 8111*). To determine the AFB1 production potential, mVOCs that are specifically emitted by strains not producing toxins will also need to be targeted: ethyl 2-methylbutyrate, β-chamigrene, α.-dehydro-ar-himachalene, α-corocalene, τ-muurolol, dodec-1-ene, 2,4,5-trimethyl-1,3-dioxolane, di-epi-1,10-cubenol, (7a-isopropenyl-4,5-dimethyloctahydroinden-4-yl)methanol, α-corocalene, and β-chamigrene. However, as some of these are emitted in low amounts, this will require the development of highly sensitive captors [39]. In this case, it could be interesting to consider the development of an electronic sensor, making the detection of productive strains in silos possible quickly and without any sample preparation [10,24,50]. The essential parameters such as selectivity and sensitivity for their design must also be taken into account [50,51,52].

Semi-quantification of terpenes showed similar amounts for the toxigenic strain ITEM 8111 and its mutant. Values ranged for day 3 from 0.1 for τ-muurolol to 8.89 ppb for β-elemene (Table 2).

The β-himachalene was detected in all our tested strains. However, its concentration was significantly higher for the non-AFB1-producing mutant (ITEM 8111*) with a peak of 2.59 ppb (against 0.74 ppb for the AFB1-producing strain) at day 3. 

Styrene is common to both toxigenic strains (ITEM 8111 and 8111*) and non-toxigenic ITEM 8088 [34]. However, the amount of styrene released could be a good indicator of the absence of AFB1 production. Indeed, 29.8 × 10^6^ ppb was released for the non-AFB1-producing mutant (ITEM 8111*), against 261.75 ppb for the AFB1-producing strain (ITEM 8111). This molecule was already detected for other fungal genera like *Penicillium sp.,* but the detected concentrations were much lower [39,49].

If the developed captors allow temporal and quantitative observations, γ-gurjunene, γ-cadinene, β-elemene, and α-selinene could act as additional indicators, as they are emitted in high proportions on the 3rd day of growth of the AFB1-producing strains (ITEM 8111). 

In order to confirm and refine the relevance of these molecules, further research is in progress on a wider variety of toxigenic and non-toxigenic strains of *A. flavus*. In vivo tests will also be needed to confirm the emission of the volatiles in real agronomical conditions. Several studies have indeed shown that mVOCs emitted by fungi vary with the substrate used [23,32].

To better understand the potential correlation between sesquiterpenes and aflatoxins production, a focus on metabolic pathways is needed. The origin of the terpene biosynthesis pathway is acetyl-CoA, which is then converted to malonyl-CoA by acetyl-CoA carboxylase. On the one hand, the combination of acetate and malonyl-CoA leads to the formation of hexanoyl units and then to norsolorinic acid, which is the first stable precursor of the aflatoxin biosynthetic pathway [53]. On the other hand, the farnesen backbone, the basis of many fungal sesquiterpenes, is derived from the isoprenoid biosynthetic pathway from the same acetyl-CoA molecule [54].

Recent studies are progressing to detect the genes involved in of each of the sesquiterpenes’ production [55], as well as studies on the aflatoxin biosynthetic pathway, which is being analyzed to better understand its functioning and genetic structure [9,56,57,58,59].

## 4. Materials and Methods

### 4.1. Fungal Strains

In order to investigate the above-mentioned hypotheses, fungal strains were provided by CNR-ISPA (Research National Council of Italy—Institute of Sciences of Food Production, Bari, Italy). The strains of *Aspergillus flavus* belong to the official collection of fungi of the Institute of Sciences of Food Production ITEM Collection, where they are available. The ITEM is recognized by the International Organization of European Culture Collections and the World Federation of Culture Collections. 

Two categories of *A. flavus* strains were studied: a non-toxigenic strain as negative control for the aflatoxin B1 (AFB1) production (designated as ITEM 8088), and a toxigenic strain which produces AFB1 (designated as ITEM 8111), as well as its mutant (ITEM 8111*), which does not produce AFB1.

### 4.2. Fungi Inoculation

Fungi were grown on SNA (Synthetic Nutriment-poor Agar) medium (for 1 L, 1 g KH_2_PO_4_; 1 g KNO_3_; 0.5 g MgSO_4_·7H_2_O; 0.5 g KCl; 0.2 g glucose; 0.2 g sucrose; 20 g agar) and stored at −80 °C in glycerol. They were incubated at 30 °C during 7 days in darkness. The spore suspensions were prepared with Tween 20 and sterile water. The concentrations were determined using a Bürker cell and adjusted to centrally inoculate 1.15 × 10^3^ spores·mL^−1^. The inoculation was carried out in 20 mL vials containing slanted PDA (Potato Dextrose Agar) to provide a larger growth surface for the fungus. The vials inoculated were incubated at 30 °C during 3, 5, 7, and 9 days in darkness before sampling. Three replicates were systematically prepared [31,43,60].

### 4.3. Aflatoxin Analysis

The aflatoxin incidence was determined using liquid chromatography coupled to mass spectrometry (LC-MS/MS) according to an in-house validated protocol. One gram of sample was taken and transferred into an extraction tube. Blanks and unknown samples were spiked with the volume as indicated in the following Table 3. The samples were left in the dark for approximately 15 min for re-equilibration. Five mL of acidified ethyl acetate (ethyl acetate + 1% formic acid, *v*/*v*) was added, and vortexed accordingly. The samples were shaken on an overhead shaker for 15 min, and centrifuged at 3600 rpm for 15 min. The supernatant was transferred onto a filter with a plastic Pasteur pipette, preconditioned with acidified ethyl acetate. Then, 5 mL of dichloromethane was added to all samples. The samples were vortexed, and centrifuged again at 3600 rpm for 15 min. Then, the supernatant was transferred onto a filter with a plastic Pasteur pipette, and preconditioned with dichloromethane. The residue was then evaporated completely in a warm water bath at 40 °C. The remaining fraction was dissolved in 200 µL of injection solvent. To fully dissolve the matrix, the dilution was vortexed for 2 min. Afterwards, 200 µL of hexane was added, and transferred to a centrifugal filter (0.22 µm). The sample was centrifuged for 10,000 rpm for 5 min, and 100 µL of the bottom layer was transferred into an LC-MS/MS vial. The samples were run according to a validated methodology, and the instrumental parameters were as described in Monbaliu et al. [61].

### 4.4. GC-MS Parameters

The volatile organic compounds (VOCs) analyses were performed on an Agilent Technologies GC 7890B fitted with a Gerstel MPS (MultiPurposeSample, (MPS, Gerstel^©^, Mülheiman der Ruhr, Germany) robotic autosampler with the SPME tool for SPME fibers modules and MSD 5977B (USA). The inoculated vials were incubated at 40 °C for 30 min and extracted for one hour at 40 °C with SPME fibers (Supelco, Darmstadt, Germany, DVB/CAR/DDMS, 50/30µm, 24 Ga). The VOCs separation was performed on an HP-5ms column (Agilent Technologies, Santa Clara, CA, USA, 5%-phenylmethylpolysiloxan, non-polar, 30 m × 0.250 mm × 0.25 µm) with a constant helium flow rate of 1.2 mL·min^−1^. The inlet SPME fibers were desorbed at 250 °C by splitless injection using an SPME inlet coating of 78.5 mm × 6.5 mm × 0.75 mm (Supelco Inc., Bellefonte, PA, USA). The temperature programs were applied as follows: 45 °C for 7 min, 5 °C·min^−1^ up to 70 °C, 70 °C for 3 min, 3 °C·min^−1^ up to 120 °C, 120 °C for 3 min, 10 °C·min^−1^ up to 270 °C, and a final hold at 270 °C for 5 min. The mass spectral analysis was performed using the electron ionization (EI) mode at 70 eV and scan mass range from 35 to 350 amu. The ion source and MS source temperatures were 250 ˚C and 280 ˚C, respectively [22,30,39,42].

### 4.5. Identification of GC-MS Analysis 

The identification was made by mass spectra comparison with NIST17 and WILEY298 libraries, and using the retention indices of Kovat (standard solution of saturated n-alkane C6-C30 (1000 mg·mL^−1^ in hexane, Supelco, Belgium)) in order to calculate the retention indices of each molecule, then using the indices associated with the Van den Dool and Kratz method. Some identifications were confirmed by injecting pure analytical standards purchased from Sigma-Aldrich (Overijse, Belgium). Some terpenes, not commercially available were confirmed by injecting in the same chromatographic conditions an essential oil (Pranarôm, Belgium) typically containing this compound as the main compound [35,62]. In this perspective, γ-gurjunene, δ-cadinene, γ-cadinene, and viridiflorol have been identified with the essential oil of *Cistus ladaniferus*; the δ-cadinene, α-selinene, α-copaene, and τ-muurolol with the essential oil of *Cedrelopsis grevei*; and finally, the β-himachalene with the essential oil of *Cedrus deodara*.

### 4.6. Statistical Model

Statistics were performed using metaboanalyst (http://www.metaboanalyst.ca, accessed on 26 march 2021) [63]. Partial Least Square Analysis (PLSDA) models were built using four components (1) to discriminate the toxigenic versus non-toxigenic strains and (2) to discriminate three classes: AFB1-producing strain, non-AFB1-producing strain, and non-toxigenic strain. For all models, the features (i.e., GCMS profiles) were log transformed and mean centered. The discrimination was visualized by plotting the first PLSDA components.

### 4.7. Semi-Quantification

In order to semi-quantify the compounds of a sample, a mixture composed of the molecules of interest as well as the five most abundant molecules present in this sample of fungi was carried out by preserving the relative area proportions between each molecule (stock solution). The standards used were bought from Sigma-Aldrich (Overijse, Belgium) when commercially available as 2-methylbutan-1-ol, 3-methylbutan-1-ol, styrene, valencene (70% purity), and heptadecane. Terpenes not commercially available were semi-quantified using valencene as a reference standard. The construction of the calibration curves was established by successive dilution of the initial mixture in ethanol (D1 = 300 µL of the stock solution, D2 = 1/2D1, D3 = 1/2D2, D4 = 150 µL of the stock solution, D5 = 1/2D4). After stirring, a volume of 1 µL of the diluted solutions was deposited at the bottom of a 20 mL vial and analyzed concomitantly as the samples [64,65,66,67,68,69,70,71,72,73,74,75,76].

## 5. Conclusions

**Figure 7:**
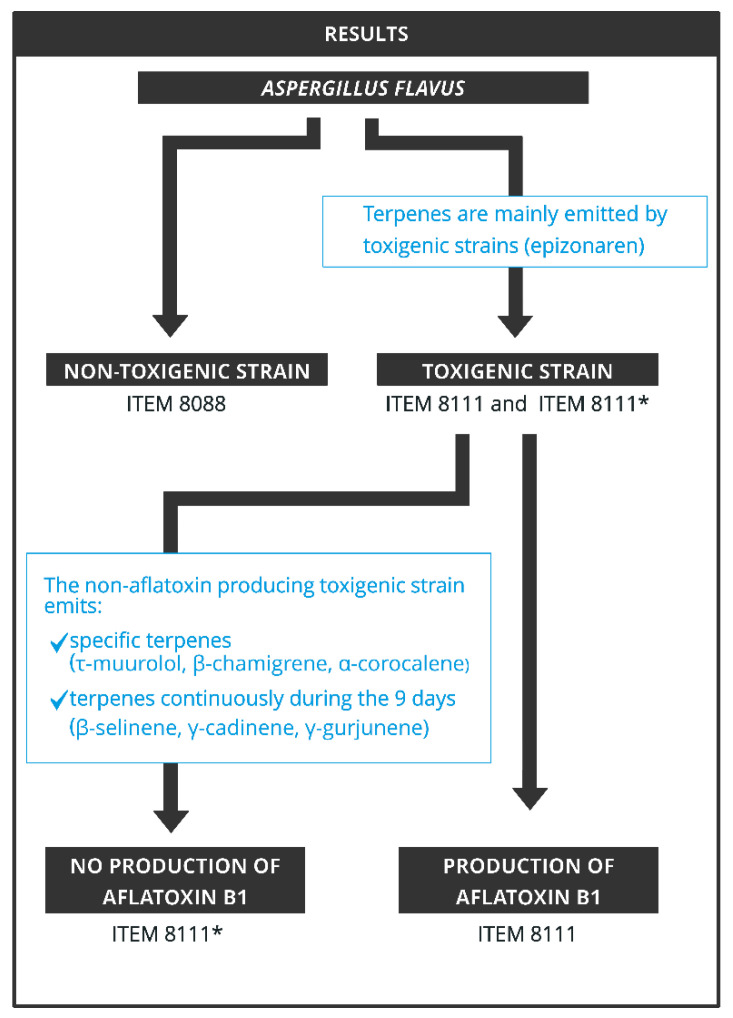
Summary of the results.

In conclusion, new mVOCS were associated with *A.flavus,* in addition to those already known in the literature to be common to *A. flavus* and other species of the genus *Aspergillus*. Comparison of non-toxigenic and toxigenic strains identified potential biomarkers, mainly terpenes, to differentiate these two categories (Figure 7). Comparison of the volatiles emitted by the toxigenic AFB1-producing strain and its non-AFB1-producing mutant surprisingly allowed the detection of a dramatic variability in terpene production between these two strains related to the lack of AFB1 production. Studies to identify genomic as well as stability assessment of this mutation that inhibited AFB1 production in the ITEM 8111* mutant strain will be performed. An approach focused on the metabolic pathways of mVOCs specific to toxigenic strains, and in particular those of certain terpenes emitted by the non-AFB1-producing toxigenic strain could be proposed in order to clarify their impact on the expression of the AFB1 biosynthesis genes, and thus determine their influence at a different scale of the fungi [58].

Finally, the semi-quantification of some molecules allowed the definition of detection thresholds for the conception of a future molecular fingerprint sensor. 

## Figures and Tables

**Table 1:** List of mVOCs present at least in two replicates. The values are the percentage of the total area. Identification by comparison with the NIST17 and Wiley298 mass spectra libraries, as well as by comparison between the literature retention index (RI (HP-5ms)), according to the method of Van Den Dool and Kratz on a non-polar HP-5ms column, and the calculated retention index (RI) for each mVOC. The retention index is established using a mixture of n-alkanes under the same chromatographic conditions.

						ITEM 8088				ITEM 8111				ITEM 8111* (Mutant)			
Name			# Cas	RI (HP-5ms)	RI	Day 3	Day 5	Day 7	Day 9	Day 3	Day 5	Day 7	Day 9	Day 3	Day 5	Day 7	Day 9
**Acetic acid**	ac	**Acid**	64-19-7	-	625	-	-	-	-	-	-	-	-	-	-	17.79	-
**2-Methylpropanoic acid**			79-31-2	785	767	-	-	-	-	-	-	-	-	-	-	-	0.51
2-Butyloctan-1-ol *		**Alcohol**	3913-02-8	-	1286	-	-	-	-	0.65	-	-	-	0.43	-	-	-
**2-Methylbutan-1-ol**	ac		137-32-6	736	720	13.2	7.84	9.32	17.8	16.25	8.87	10.5	10.5	32.21	16.84	11.34	11.35
**2-Methylpropan-1-ol**	bc		78-83-1	622	624	11.39	27.10	37.01	40.07	26.67	32.76	50.92	29.47	27.10	25.08	36.50	35.68
**3-Methylbutan-1-ol**	ac		123-51-3	734	724	13.3	11.6	11.9	14.1	13.09	13.04	8.48	16.6	21.18	11.54	8.57	9.11
**Butan-1-ol**	ab		71-36-3	668	648	-	-	-	-	-	-	-	-	-	8.57	9.45	9.30
**Butan-2,3-diol**	abc		513-85-9	804	809	-	0.37	0.43	-	-	-	1.04	-	-	-	-	-
**Butan-2,3-diol (enantiomer)**	abc		24347-58-8	-	816	-	-	-	-	-	-	0.41	-	-	-	-	-
**Decan-1-ol**	b		112-30-1	1272	1272	-	-	-	-	0.53	-	-	-	0.36	-	-	-
**Ethanol**	abc		64-17-5	-	575	94.95	100	100	90.40	100	100	80.97	97.52	62.03	100	100	100
**Propan-1-ol**	abc		71-23-8	-	595	-	26.2	31.4	-	36.42	39.75	13.4	33.04	51.76	14.77	37.05	34.78
**Propan-2-ol**	a		67-63-0	-	584	-	-	-	72.3	-	-	-	-	-	-	-	-
**2-Methylbut-2-enal**		**Aldehyde**	497-03-0	737 *	723	4.22	2.19	3.39	-	4.30	3.14	5.84	6.70	3.62	-	-	-
**2-Methylbutanal**	abc		96-14-0	660	649	12.54	12.64	15.16	22.1	10.26	10.99	10.03	17.09	9.77	-	-	-
**3-Methylbutanal**	abc		590-86-3	649	643	5.21	5.76	-	-	-	10.01	7.32	11.77	-	-	-	-
**Acetaldehyde**	ab		75-07-0	-	566	-	-	-	-	-	-	6.82	-	-	-	-	-
(E,Z)-1,2-diethylidenecyclopentane*		**Alkane**	Not available	-	975	-	-	0.36	-	-	-	-	-	-	-	-	-
**2,2,4,6,6-pentamethylheptane**	b		13475-82-6	997	984	1.22	-	-	-	0.52	0.63	-	-	-	-	-	-
4,6-Dimethyldodecane *			61141-72-8	-	1277	-	-	-	-	0.57	-	-	-	-	-	-	-
**Heptadecane**	bc		629-78-7	1700	1696	-	-	-	-	1.39	-	-	-	1.61	0.80	0.47	0.54
**Heptane**	ac		142-82-5	700	677	1.57	0.26	0.10	-	-	-	-	0.48	-	-	-	-
**Hexane**	c		110-54-3	600	612	4.68	17.18	-	8.62	-	17.97	-	13.93	-	-	-	-
Methyl-cyclooctane *	c		1502-38-1	-	1386	-	-	-	-	-	-	1.49	-	-	-	-	0.33
Nonyl-cyclopropane *			74663-85-7	-	1273	-	-	-	-	0.49	-	-	-	-	-	-	-
**Octane**	c		111-65-9	800	788	-	-	-	-	-	-	-	1.76	-	-	-	-
Bicyclo[2.2.0]hexa-2,5-diene *		**Alkene**	7641-77-2	-	1380	-	-	-	-	-	-	-	-	-	-	-	0.33
**Dodec-1-ene**			1120-36-1	1187	1188	-	-	-	-	-	-	-	-	0.50	-	-	0.27
**Styrene**	abc		100-42-5	898	882	8.31	-	-	6.10	3.73	-	-	-	63.64	42.46	32.57	29.06
**Toluene**	abc		108-88-3	762	745	-	-	-	-	-	-	-	2.37	-	-	-	-
**Ethyl 2-methylbutyrate**	bc	**Ester**	7452-79-1	-	840	2.28	-	0.60	2.84	-	-	-	-	-	-	0.31	0.35
**Ethyl acetate**	bc		141-78-6	612	618	5.87	12.9	18.7	17.1	3.62	14.5	-	12.7	11.93	9.00	13.08	11.67
**Ethyl butyrate**	c		105-54-4	802	795	-	-	-	-	-	-	-	0.36	-	-	-	-
**Ethyl isobutyrate**	c		97-62-1	-	740	1.01	0.42	1.20	2.09	0.41	0.31	-	1.28	1.51	0.62	0.25	0.43
**Ethyl phenylethanoate**	c		101-97-3	1248	1242	-	-	-	-	-	-	-	-	-	-	-	0.39
**Ethyl propanoate**	c		105-37-3	714	695	1.66	-	-	0.32	0.57	-	-	0.30	-	0.64	0.54	0.42
**2,5-Dimethylfuran**	c	**Furan**	625-86-5	-	689	0.75	-	0.14	0.21	-	-	-	0.15	-	-	0.25	0.27
**2-Methylfuran**	abc		534-22-5	603	615	-	-	-	-	-	-	10.71	-	-	-	-	-
**3-Hydroxybutan-2-one**	c	**Ketone**	513-86-0	-	695	-	0.37	0.40	-	-	2.03	1.26	-	-	-	-	-
**Butan-2-one**	a		78-93-3	605	609	-	-	-	-	-	-	-	-	23.00	12.42	-	19.29
Thiochroman-4-one *			3528-17-4	-	1124	-	-	-	-	-	-	-	-	0.56	-	-	-
**NI 640**		**Other**	-	-	640	-	-	12.7	-	-	-	-	-	-	-	-	-
**NI 729**			-	-	729	-	-	1.74	-	-	-	-	-	-	-	-	-
**NI 756**			-	-	756	-	-	0.11	0.47	-	-	-	-	-	-	-	-
**NI 1271**			-	-	1271	-	-	-	-	-	-	-	-	0.41	-	-	-
**NI 1323**			-	-	1323	-	-	-	-	0.46	-	-	-	-	-	-	-
**NI 1501**			-	-	1501	-	-	-	-	-	-	-	-	0.45	-	-	-
2,4,5-Trimethyl-1,3-dioxolane *			3299-32-9	752 *	708	-	-	0.32	-	-	-	0.31	-	-	-	-	-
**Trichloromethane**	c		67-66-3	-	623	3.76	-	-	-	8.20	-	-	-	-	-	-	-
4a,8-Dimethyl-2-(prop-1-en-2-yl)-1,2,3,4,4a,5,6,7-octahydronaphthalene *		**Terpene**	103827-22-1	-	1476	-	-	-	-	-	-	-	-	-	0.46	0.68	0.57
(7a-Isopropenyl-4,5-dimethyloctahydroinden-4-yl)methanol *			Not available	-	1738	-	-	-	-	-	-	-	-	0.54	-	-	-
**Di-epi-1,10-cubenol**			73365-77-2	1623	1611	-	-	-	-	-	-	-	-	-	-	-	0.16
**Aromadendrene**	c		109119-91-7	1444	1443	-	-	-	-	0.86	-	-	-	0.70	-	-	-
cis-Muurola-3,5-diene *	b		157374-44-2	1447 *	1448	-	-	-	-	2.43	-	-	-	1.97	-	0.34	0.26
**Epi-bicyclosesquiphellandrene**	abc		54274-73-5	1478	1463	-	-	-	-	6.27	-	-	-	4.04	1.25	0.85	0.68
Epi-cubeno-1-ol *			19912-67-5	1619 *	1611	-	-	-	-	0.94	-	-	-	0.79	-	-	-
**Epizonaren**	abc		41702-63-0	1497	1494	-	-	-	-	23.22	6.38	4.22	1.27	17.54	7.17	5.15	4.50
**Germacrene-D**	ab		23986-74-5	1480	1480	-	-	-	-	4.24	0.63	-	-	3.03	0.92	0.59	0.46
**Palustrol**			5986-49-2	1569	1565	-	-	-	-	0.34	-	-	-	0.45	-	-	-
**trans-Caryophyllene**	abc		87-44-5	1418	1414	-	-	-	-	1.63	-	-	-	1.12	-	-	-
**Valencene**	abc		997297	1490	1491	-	-	-	-	-	-	-	-	-	-	-	0.69
**Viridiflorol**			552-02-3	1589	1589	-	-	-	-	0.51	-	-	-	0.74	-	-	-
**α.-Dehydro-ar-himachalene**			78204-62-3	1522	1537	-	-	-	-	-	-	-	-	0.36	-	-	-
**α-Cadinene**	b		24406-05-1	1538	1534	-	-	-	-	1.59	-	-	-	0.78	0.24	-	0.09
**α-Cadinol**			481-34-5	1656	1654	-	-	-	-	-	-	-	-	0.53	-	-	-
**α-Calacorene**	b		21391-99-1	1540	1540	-	-	-	-	0.55	-	-	-	0.56	-	-	-
**α-Copaene**	ac		3856-25-5	1372	1365	-	-	-	-	1.16	-	-	-	1.20	-	-	-
**α-Corocalene**	bc		20129-39-9	1629	1620	-	-	-	-	-	-	-	-	0.32	-	-	-
**α-Cubebene**	a		17699-14-8	1348	1342	-	-	-	-	0.69	-	-	-	0.79	-	-	-
**α-Gurjunene**	ac		489-40-7	1408	1401	-	-	-	-	2.02	-	-	-	1.45	-	0.26	0.22
**α-Isocomene**			65372-78-3	1392	1380	-	-	-	-	3.51	0.47	-	-	2.21	0.65	0.40	-
**α-Muurolene**	bc		31983-22-9	1472	1471	-	-	-	-	0.88	-	-	-	0.64	-	-	-
**α-Selinene**	ab		473-13-2	1494	1491	-	-	-	-	6.73	-	-	-	4.73	1.42	0.88	-
**β-Cadinene**	c		523-47-7	-	1489	-	-	-	-	1.11	-	-	-	0.72	-	-	-
**β-Chamigrene**	a		18431-82-8	1472	1476	-	-	-	-	-	-	-	-	1.05	-	-	-
**β-Elemene (E)**	abc	**Terpene**	515-13-9	1382	1376	-	-	-	-	1.85	-	-	-	0.93	-	3.05	1.83
**β-Elemene (Z)**	abc		515-13-9	1382	1384	-	-	-	-	35.71	4.15	-	-	17.10	5.13	-	-
**β-Himachalene**	abc		1461-03-6	1498	1497	0.54	-	-	-	2.53	1.47	-	-	6.41	5.24	4.46	5.61
**β-Selinene**	a		17066-67-0	1479	1483	-	-	-	-	3.30	-	-	-	2.35	0.65	0.42	0.28
**γ-Cadinene**	abc		39029-41-9	1513	1508	-	-	-	-	18.85	1.93	-	-	9.95	3.30	2.09	1.57
**γ-Gurjunene**	ac		22567-17-5	1476	1472	-	-	-	-	10.06	-	-	-	6.37	1.66	1.17	0.95
**γ-Muurolene**	abc		30021-74-0	1477	1477	-	-	-	-	2.80	-	-	-	0.99	-	-	-
**δ-Cadinene**	abc		483-76-1	1524	1520	-	-	-	-	26.06	5.91	2.92	0.92	18.11	6.58	4.43	3.68
**τ-Muurolol**			19912-62-0	1641	1644	-	-	-	-	-	-	-	-	0.38	-	-	-

NI: not identified, *: potentially identified based on the mass spectra libraries or retention index only, a: compound listed as being of filamentous fungal origin, b: compound listed as being of the genus *Aspergillus*, c: compound listed as being of the species *Aspergillus flavus* in accordance with the literature [10,23,28,29,30,31,32,33,34,35,36,37,38,39,40,41,42].

**Table 2:** Compounds quantification (ppb) emitted by the strains on day 3.

Compound	ITEM 8111	ITEM 8111*
α-Cadinene	0.432	0.277
α-Cadinol	-	0.175
α-Isocomene	0.950	0.720
α-Muurolene	0.282	0.209
α-Selinene	1.817	1.565
β-Chamigrene	-	0.370
β-Elemene	8.897	5.181
β-Himachalene	0.737	2.590
δ-Cadinene	6.042	7.874
γ-Gurjunene	2.615	1.895
γ-Muurolene	0.769	0.381
τ-Muurolol	-	0.105
Aromadendrene	0.205	0.255
Epi-cuben-1-ol	0.311	0.360
Epizonaren	7.128	5.948
Germacrene-D	1.132	0.996
Styrene	261.75	29.8 × 10^6^
2-Methylbutan-1-ol	2.223	0.888
3-Methylbutan-1-ol	0.934	0.440

**Table 3:** Treatment of blank and unknown samples for LC-MS/MS analysis.

Samples	Aflatoxin Mixture 2.5 ng·µL^−1^	Zearalanone 10 ng·µL^−1^	Deepoxydeoxynivalenol 50 ng·µL^−1^
Blank	-	20	10
Spike 0.5 X	10	20	10
Spike 1 X	20	20	10
Spike 1.5 X	30	20	10
Spike 2 X	40	20	10
